# Using Blockchain to Create Transaction Identity for Persons Experiencing Homelessness in America: Policy Proposal

**DOI:** 10.2196/10654

**Published:** 2019-03-06

**Authors:** Anjum Khurshid, Ashish Gadnis

**Affiliations:** 1 Dell Medical School The University of Texas at Austin Austin, TX United States; 2 BanQu, Inc Austin, TX United States

**Keywords:** affordable housing, Austin, blockchain, distributed ledger, emergency medical services, health information, homelessness, interoperability, transaction identity

## Abstract

More than 500,000 people experience homelessness in America each day. Local and federal solutions to the problem have had limited success because of the fragmentation of services and lack of valid and timely information. Billions of dollars spent to provide reliable, timely, and actionable information in health care have exposed the difficulty of establishing such a system using the prevalent information technology solutions. However, relying on successful examples of the use of blockchain to help refugee populations and poor farmers internationally, we have partnered to propose an innovative solution to this problem using the case of people experiencing homelessness in Austin, Texas. This paper aims to describe one of the first applications of blockchain technology for addressing homelessness in the United States by creating a digital identity for people experiencing homelessness and engaging emergency medical services and clinical providers. The authors argue that a lack of documentation to prove personal identity and the inability to access own records are major hurdles for empowering persons experiencing homelessness to be resilient and overcome the life challenges they face. Furthermore, it is argued that this lack of information causes misdiagnosis, duplication, and fragmentation in service delivery, which can be potentially addressed by blockchain technology. Further planning for creating a program on the ground with additional funding will demonstrate the results of using blockchain technology to establish digital identity for persons experiencing homelessness.

## Introduction

Over 500,000 people in America suffer from homelessness every night [1]; of these, 37% are children, 8% are veterans, and 48% are disabled. As cities across the United States face the ever-visible unsheltered population of homeless on its streets, the solutions are mostly about throwing more money and resources to address the symptoms rather than the underlying causes. Austin, Texas, is one of the fastest growing cities in the United States [2]. Although it faces similar issues of homelessness as any other city, it has the opportunity to test a disruptive technology, like blockchain, to develop a more effective and efficient solution to this chronic problem. This paper aims to describe the concept of how such technology innovation may work and to explain the solution proposed by a collaboration of city leaders, health and social service agencies, technology experts, and population health researchers.

## The Homelessness Challenge

There is a general concern about homelessness in America owing to a growing shortage of affordable rental housing and a simultaneous increase in poverty. Income inequality and institutional discrimination exacerbate the situation. The National Coalition for the Homeless lists poverty, lack of affordable housing, job loss, lack of health care, mental illness, substance abuse, and domestic violence as leading factors for homelessness. The report “Discrimination and Economic Profiling among the Homeless of Washington DC” 2014 showed the experiences of individuals living in homelessness and the discrimination they face. Reportedly, 42% are African American, 31% are non-Hispanic white people, and 24% are Latino people [3].

The situation of homelessness in Austin is quite grim (Table 1). On any single day, >2000 people experience homelessness in Austin. Annual figures range from 7000 counted homeless to >12,000 estimated people. Of those accounted for, only one-third sleep in a shelter every night, while a quarter sleep on the street sidewalk or a doorway, 16% sleep in a park, 13% sleep in a vehicle, and 9% sleep under bridges and in abandoned buildings. A census of persons experiencing homelessness showed that 80% are unemployed or have no earned income, 60% report a problem with drugs or alcohol in their lifetime, and 45% have a current mental health problem [4].

In addition, persons experiencing homelessness in Austin face many health challenges, which lead to overutilization of more expensive services like emergency departments and hospitalizations. This inefficiency adds to the overall cost of providing health care in the city and increases the burden on taxpayers. Of note, 60% of the homeless surveyed in Austin in 2015 had a drug or alcohol problem at some point in their lifetime, and 38% reported having been treated for it recently. Moreover, 48% suffered from mental health issues, and most of them had an ongoing issue that resulted in hospitalization. About 25% suffered from a chronic condition; one in five people reported a history of hepatitis C infection, and one in four people had a history of heat stroke or exhaustion [4].

Furthermore, the homeless are high users of health services, with >62% reported being in the emergency room in the past 6 months. Notably, 40% had used an ambulance to go to a hospital, and almost one-third had been hospitalized. Many used the hospital as their main point of access to get care. It is estimated that these high users of services cost the taxpayers about US $222,000 per person annually only for hospital care, emergency room visits, and emergency medical services transport [4].

**Table 1:** Profile of homeless in Austin and Travis County [3,4.

Demographics		Homeless in the county (n=7100 as per HMIS^a^ census 2015), %	General population in the county (n=1,226,000 as per US Census Bureau 2016), %
**Age (years)**			
	0-17	9	23
	18-24	5	10
	25-44	33	36
	45-64	40	23
	≥65	3	8
**Race or ethnicity**			
	African American	42	8
	Non-Hispanic white	31	50
	Latino	24	34
	Other	3	8

^a^HMIS: homeless management information systems.

## The Underlying Causes of Homelessness

If providing affordable public housing and timely health care costs only a fraction of the cost incurred by public resources for a homeless person ($40,000 vs $222,000 per person annually) [4], the programmatic intervention to reduce homelessness seems straightforward. However, the existing social, health, and economic benefit systems in the public sector have limitations on how they may effectively help individuals who experience homelessness. Most policy solutions for homelessness focus on developing affordable housing units, employment assistance, or medical care programs, and few provide an integrated approach for delivering these services [5]. The public programs are usually organized around administrative departments in the city or county government rather than around the needs of individuals. If the solution is to be developed from a person-centered approach, then one of the key challenges of implementation is the system’s inability to accurately collect, share, and verify information about the person experiencing homelessness. In the absence of verifiable information, the services are fragmented and an individual is unable to apply and benefit from any of the available services without going through multiple, duplicative, and bureaucratic requirements [5]. Such inefficiency is an unnecessary burden on the budgets of service providers and on the taxpayers who fund such public programs.

As far as information sharing is concerned, we may learn from our national experience in health information technology. Meaningful information sharing of patient data for delivery of health services has turned out to be a nontrivial task [6]. This is a problem of not only the poor and uninsured patients only, but those with college education, employer-based health insurance, and above-median household incomes also suffer the consequences of the lack of information sharing in health care and social services. Billions of dollars spent on interoperability and “meaningful use” of health information technology systems have not been able to improve effective health care data sharing [7,8]. Whether it is owing to lack of incentives, structural problems of the system, or limited access to personal information by patients, it is unlikely that the required level of seamless information sharing in health will be achieved in the near future. The federal health information technology policy in the past 10 years clearly shows that while more resources can help in digitizing medical records, success in the effective use of the information cannot be accomplished with incomplete and marginal solutions [9,10]; it requires fixing the structural deficiencies of the health system. If the past is any indication of the future of health system transformation, it will be a long time before these structural issues around information sharing, patient empowerment, and value-based incentives will be resolved. Therefore, it can be argued that achieving information sharing through a person-centered approach to homelessness by using traditional information technology strategies is not likely to deliver results.

In contrast to the US experience in health information technology, there are some examples of innovative solutions that have been successfully applied to address the information-sharing problem in health and social systems outside the United States. International programs in resource-constrained economies may help us gain useful insights because they are generated in an environment of poverty and lack of resources [11,12]. The use of information and communication technologies to deliver health and other social services has been innovative and quite successful in many developing countries. The ubiquitous availability of internet connectivity and a new way of managing information and trust using blockchain technology seem to provide better hope for integrating information about an individual from different sources and solving the problems with information sharing described above [13-15].

## Blockchain Technology’s Promise

**Figure 1:**
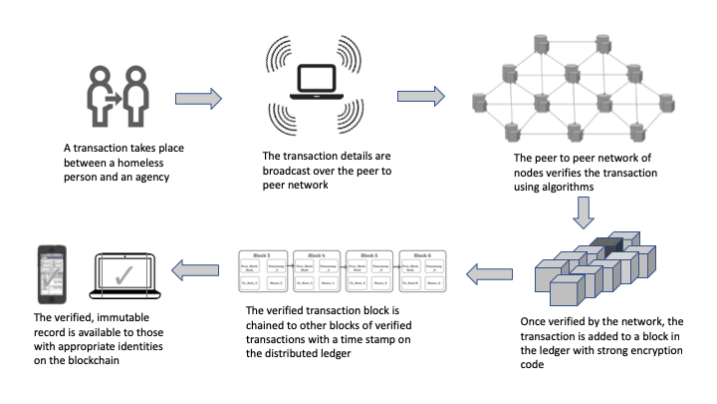
A simplified version of how Blockchain’s distributed ledger works.

Blockchain technology has been touted to disrupt social and economic systems in society like the internet did [16,17]. Recent spikes in the value of bitcoin cryptocurrency is only a glimpse of how the market is gauging its potential in the future [18,19]. However, despite the hype about bitcoin and other cryptocurrencies, the often overlooked fact is that the real value of this experience lies in its underlying technology—blockchain based on distributed ledger technology (DLT). Blockchain can be described in many technical ways; yet, the simplest nontechnical definition is technology that uses DLT as a distributed data network, whereby all parties in a transaction get a secured and immutable copy of that transaction [20,21]. Figure 1 presents a simplified explanation of the DLT.

Blockchain technology is a new way of managing information, assets, and identity. It uses secure, immutable, append-only, timestamped content that is distributed over a network, which makes it almost impossible to hack [22]. The reason it is being hailed as such a disruptive technology is because it (1) takes out the intermediaries in transactions by creating a trust network among users; (2) promises a future that is truly based on distributed architecture without large central databases; (3) takes back control of an individual’s data from other organizations and businesses and gives it to the individual; (4) makes all data highly liquid and portable, making geographical and political boundaries irrelevant; and (5) stores every transaction in multiple locations and minimizes hacking or fear of loss of data.

## Application of Blockchain Internationally

Lack of “transaction identity” is a concept that provides a possible explanation of how people in poverty, refugees, or homeless people are prevented from breaking the cycle of being a beneficiary of available support services by public and private agencies. “Transaction identity” is defined as a collection of key aspects and relevant interactions that build a person’s history and standing in society [23].

There are many examples of how the underlying technology of distributed ledgers in blockchain is being used internationally to solve the issues of transaction identity in poorer countries (Textbox 1; Figure 1). These projects rely on DLT developed for international humanitarian programs. The unique aspect of this implementation is that it deploys DLT or blockchain without any link to cryptocurrencies, like bitcoin, thereby avoiding some of the most notorious use cases of this technology. The DLT-based platform has been a mobile-friendly, affordable, and effective way to help refugees in the Middle East, poor farmers in South America, and HIV patients in Africa to establish their transaction identities, connect them to their records, and actively participate in global economic and social systems [24].

These global examples of the use of blockchain technology to address humanitarian use cases without exposing the vulnerable populations to cryptocurrency volatility and data-hacking risks may help in understanding the relevance of DLT to homelessness in America. One unique application of blockchain technology is to provide end-to-end visibility and proof of delivery of pharmaceutical products in developing countries in partnership with a global pharmaceutical company. For instance, an international supply chain problem is that of pilferage in pharmaceutical aid to places where tracking is difficult, and the aid is delivered to those who are not connected to global identity systems. When blockchain identities are generated for the commodities being shipped through intermediary suppliers in the global system and eventual beneficiaries, the immutability and distributed ledger entries prevent any fraud or reporting errors. Furthermore, the system tracks delivery to individuals whose identities are verifiable on the blockchain platform [25].

Another example is that of microenterprise solutions to pay farmers in Latin America by creating an economic identity of farmers on a blockchain platform [26]. Farmers usually do not have bank accounts and hence have to rely on middlemen to get paid for their harvests. This puts farmers at the mercy of intermediaries who maximize their profits by negotiating minimum payments for the produce from these farmers. By removing the need of intermediaries and helping create an economic identity of these farmers who can now sell their produce directly to buyers and get paid directly through mobile payments, the blockchain technology improves the economic condition of farmers and brings them into a global economy.

The exciting feature of these international examples is that all they need is a mobile phone or internet connectivity to help marginalized community members in resource-poor environments to establish and take control of their identity without the need of an intermediary. In addition, their identity allows them to link their disparate economic and social data through blockchain technology into an immutable and verifiable global information system. The examples above can be extrapolated to any other sector where information and data need to be shared, such as health, education, financial assistance, or microenterprise loans. It is therefore not a stretch of the imagination to consider the same DLT platform, which has been applied to refugees and farmers, to be used to connect social and health data of those experiencing homelessness in Austin.

**Example of how the underlying technology of distributed ledgers in blockchain is being used internationally to solve the issues of transaction identity in poorer countries.**

Blockchain application is live in multiple countries in the following use cases:

1. Last mile transparency and traceability in supply chains for farmers to end a form of modern-day slavery
2. Education and health records access and ownership for refugees and migrant workers
3. Enabling gender equality for women farmers via access to better financial inclusion

## Austin Blockchain Project for the Homeless

A practical way to understand the application of the DLT platform using blockchain, described above, is to take the case of a person experiencing homelessness on the streets of Austin. Persons experiencing homelessness in Austin today, unfortunately, have a very disjointed and often error-prone experience when it comes to health care [27].

This process is quite familiar to anyone who works in health care, or for that matter, in any other social service sector (Textbox 2). Persons experiencing homelessness in Austin receive services from multiple agencies that keep their beneficiaries’ records in their organization’s information silos [28]. Neither the person nor anyone outside each agency has the ability to access relevant information and coordinate the needs of the person experiencing homelessness in any systematic fashion.

Almost every major city is working on improving this system of data sharing for people experiencing homelessness, and many have created homeless management information systems (HMIS) [29]. Although it is an important part of the data ecosystem for persons experiencing homelessness, the HMIS may only allow better case management and tracking but may not ensure coordination of services and involvement of the beneficiaries of services. If each encounter and transaction of a person experiencing homelessness is recorded, validated, shared, and available in one place, we can solve the problem of information sharing, coordination, and validation of identity (Textbox 3). The development of a blockchain platform as a system to capture these transactions may be achieved in a fraction of the time it will take to build an interoperable information system with interfaces among disparate systems. Furthermore, the blockchain system will be a truly person-centered, distributed, and authenticated information system that is, otherwise, extremely difficult and costly to develop under traditional information system designs.

**A basic flow of the reality that people experiencing homelessness face every day.**

1. A person experiencing homelessness in Austin downtown has a medical or mental health event that requires care.
2. 911 is called, and Austin emergency medical services (EMS) picks up the person.
3. Not having any record for this person’s prior health conditions, EMS takes the person to the local emergency department.
4. There is no historical information about the person in the hospital’s record as well because there is no driver’s license or government-issued identity available.
5. As the person experiencing homelessness is cared for in the emergency department, the lack of prior medical history puts the patient at risk for receiving wrong medications, misdiagnosis, and duplicative testing/imaging.
6. When discharged after treatment, the person cannot understand or recall all the transactions and treatments that occurred during the hospital visit; this information is also not shared with anyone outside the emergency department.
7. A few days or weeks later, Austin EMS is again asked to pick up the same person from the street to a different hospital in the city that is not linked to the first hospital.
8. The same process is repeated, with even more chances of duplication, error, and overtreatment; yet, the patient still possesses no details of this encounter and no one outside the second hospital emergency department has that information for future use.

**Steps required to change this system using traditional technology solutions.**

1. Changing the processes to capture and store data in a standardized way, so it can make sense to those outside the immediate care provider.
2. Developing linkages, usually technical pathways, to send and receive data from different data systems.
3. Understanding and mapping how data are stored in different organizations and what their terms mean (semantic interoperability).
4. Agreeing on protocols for sharing information at a technical level.
5. Creating a data governance structure, so data are used for the purposes they were shared by individuals. In many cases, a person’s consent is required to share information, which needs a separate consent management strategy.
6. At every step, there is a workflow change that is to be managed, and people who are affected by it need to be convinced of the need for change. Sometimes, further training is required to implement these changes.
7. The system of data sharing has to be tested and constantly validated, as new information may overwrite previously stored data, causing new discrepancies across different data systems.

## Austin’s Innovation Using Blockchain Technology

The city of Austin and the Dell Medical School’s Population Health Department have partnered to test a blockchain data-sharing approach in Austin to coordinate services for those experiencing homelessness. As an academic institution, the Dell Medical School has a unique mission to improve the health of the community by creating a vital and inclusive ecosystem [30]. As a 21st-century medical school in a tier 1 research university, the Dell Medical School is geared to provide disruptive solutions to persistent health problems, and homelessness is one of them.

The idea is to begin by building trust relationships with a select number of people living on the streets of Austin. The program will use a DLT-based platform to create profiles for persons experiencing homelessness, using biometric features. This allows individuals to have direct access to all transactions that are recorded on the platform through any interaction in the system while also allowing each agency to access that record with permission of individuals (Textbox 4). The solution does not rely or restrict itself to any specific platform because all the information will be on a blockchain ledger and available with the consent of an individual. Simultaneously, the service providers can create their profile or accounts on the DLT platform as well. This will include a clinical site, emergency medical services, and a local homeless coalition organization. As a person experiencing homelessness touches any of these agencies, they exchange the encounter on a ledger that is shared between their accounts (the service provider and the client).

**Figure 2:**
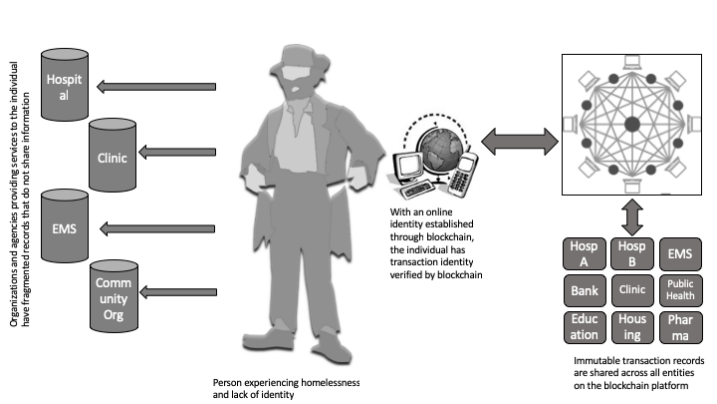
Austin blockchain project for homelessness. EMS: emergency medical services; Pharma: pharmacy; Hosp: hospital.

There will be a secured, immutable identity created for every individual in the program. This identity will allow persons to access their health data and details of the health encounter without violating any Health Insurance Portability and Accountability Act of 1996 rules. Next time the same person comes back for services or goes to another agency, the same process is repeated. As the number of these encounters increase, a copy of the transaction or encounter is recorded on the ledger that is on the person’s account and available to that person at any place or any time. For example, when this person goes to the emergency department of a new hospital, the providers can see the history of encounters with other health care providers in the past as soon as the person gives them access to share the ledger (Figure 2).

The solution solves multiple existing problems about the lack of coordination and data sharing, difficulty in implementing “no-wrong-door” policies, duplication, rising programmatic costs, absence of person-centered care focus, and challenges with the portability of records in a highly mobile population. The flexibility and universality of the blockchain platform allow individuals to use any blockchain technology to claim their identity and transactions in the future.

**Steps for the blockchain program.**

1. Persons experiencing homelessness, with no identity or incomplete health and financial records, are connected to a blockchain app through their phone.
2. Health and social service providers connect to the blockchain app to create their identities and become part of the blockchain network.
3. Persons experiencing homelessness, with their identity on the platform, are connected to the service providers on blockchain and can access those data through a mobile phone or computer.
4. Transactions at hospitals and clinics and with emergency medical services and other city agencies are recorded on the blockchain and then available to the person through the blockchain app.

## Conclusion

This paper describes a few examples of how blockchain’s DLT is being applied to create transaction identity for farmers, refugees, and microenterprise owners. The technology allows these people to become part of a distributed information and economic system and establish their identities through a secure, immutable, portable, and mobile system. In addition, it integrates all transaction data for an individual in this system to be available when needed. The replication of this technology platform for connecting social and health service providers of people experiencing homelessness is a novel and disruptive technology application. The Dell Medical School and the city of Austin are partnering to test this blockchain technology to connect service providers, including hospitals and clinics, and emergency medical services of the city. The proposal is for individuals experiencing homelessness to create an identity on a blockchain platform and to be able to record all their transactions with health providers and emergency medical services through the same ledger. The health providers and any social service agency serving the people experiencing homelessness will also need to become part of the transaction network.

Using blockchain technology solves many unsolvable problems in health and homeless care; it is a low-cost solution and does not require millions of dollars spent on this problem. In addition, it does not need building of expensive infrastructure to maintain databases defined by organizations and public departments in silos. Instead, it allows individuals to maintain all information related to their financial, health, or social history on the blockchain; it also connects providers without the expenditure of huge amounts of money to secure records. The Austin experiment of using blockchain technology for establishing transaction identity of persons experiencing homelessness has the potential to open avenues for solving this issue across the country and even globally.

## Abbreviations

DLT: distributed ledger technology
EMS: emergency medical services
HMIS: homeless management information systems
